# Plasmodium falciparum non-synonymous Kelch13 mutations mediating artemisinin resistance in East Africa: A systematic review and meta-analysis: 2014–2024

**DOI:** 10.1371/journal.pone.0354429

**Published:** 2026-07-28

**Authors:** Anthony Kapesa, Vito Baraka, Richard Mwaiswelo, Maria Zinga, Karol J. Marwa, Eliningaya J. Kweka, Erasmus Kamugisha

**Affiliations:** 1 Department of Community Medicine, School of Public Health, Catholic University of Health and Allied Sciences, Mwanza, Tanzania; 2 National Institute for Medical Research, Tanga Centre, Tanga, Tanzania; 3 Department of Microbiology, Immunology, and Parasitology, Kairuki University, Dar es Salaam, Tanzania; 4 Department of Parasitology and Medical Entomology, Catholic University of Health and Allied Sciences, Mwanza, Tanzania; 5 Department of Pharmacology, School of Medicine, Catholic University of Health and Allied Sciences, Mwanza, Tanzania; 6 Pesticides Bioefficacy Section, Department of Pesticides Management, Tanzania Plant Health and Pesticides Authority, Arusha, Tanzania; 7 Department of Biochemistry, School of Medicine, Catholic University of Health and Allied Sciences, Mwanza, Tanzania; Department of Medical Research (Lower Myanmar) Advanced Molecular Research Center, MYANMAR

## Abstract

**Background:**

Resistance to antimalarial drugs has posed a significant challenge to global efforts to control and eliminate malaria. Partial artemisinin resistance has been observed in East Africa, a region that has been a historical hotspot for antimalarial drug resistance across the continent. Consequently, this review assesses the extent of non-synonymous mutations mediating artemisinin resistance, the varieties of these mutations, and their effects on treatment outcomes in East Africa.

**Methods:**

Studies reporting artemisinin resistance (samples collected between 2014 and 2024), particularly the *Pf-Kelch13* mutation among malaria patients in East Africa, were searched through the Medline, Cochrane Central Register of Controlled Trials (CENTRAL), LILACS, and EMBASE online databases. The protocol for the review was registered at PROSPERO (Reference number: CRD42024602752). Two independent reviewers extracted data. Potential publication bias was assessed using a funnel plot. Pooled proportion estimates were calculated using a random-effects model, and heterogeneity was assessed using I^2^ statistics.

**Results:**

Twenty-four (24) studies were deemed eligible for data extraction. The heterogeneity among the studies included in the meta-analysis was high (I^2^ > 95% and p < 0.01). The overall estimated pooled proportions of non-synonymous *Pf-Kelch13* mutations, using the random effects model, were 5.0% (95% CI 3.0%–7.0%), with the pooled proportion estimates being higher in Rwanda and Uganda (10.0%, 95% CI 4.0%–16.0%) and (10.0%, 95% CI 6.0%–14.0%), respectively. Subgroup analysis (per mutation type) revealed that R561H and A675V were the most prevalent non-synonymous mutations (9.0%, 95% CI 5.0%–15.0% and 7.0%, 95% CI 4.0%–10.0%, respectively). Patients harbouring parasites with *Pf-Kelch13* non-synonymous mutations were significantly more likely to experience treatment failure than those harbouring wild *P. falciparum* parasites (Log OR: −2.06, 95% CI, −2.71–1.41).

**Conclusion:**

The prevalence of *Pf-Kelch13* non-synonymous mutations known to be associated with artemisinin resistance was significant. The most common mutations identified were R561H and A675V. Continued molecular surveillance and coordinated efforts are essential to contain the partial artemisinin resistance in the East African region and prevent its spread across the continent.

## Background

Despite the substantial progress made in sub-Saharan Africa (sSA), particularly in East Africa, the reduction of malaria transmission has recently stagnated [1]. In general, the current malaria control and elimination efforts confront challenges due to insufficient funding, emerging vector resistance, and the looming threat of artemisinin resistance [2]. The recent emergence and spread of partial artemisinin resistance in the East African region is concerning [3], as it could further compromise the progress made in reducing the disease burden in the region and Africa at large.

Previous evidence showed that resistance to the primary antimalarials, chloroquine (CQ) and later sulfadoxine-pyrimethamine (SP), spread from SEA to the East African region and throughout Africa; thus, EA is a historical hotspot for antimalarial resistance in Africa [4,5]. However, recent evidence on artemisinin records a change in trend whereby the *Pf*-*Kelch13* mutations identified in the East African region have been confirmed to be indigenous (do not relate to those reported in SEA [3,6]. Partial artemisinin resistance was first recorded in Rwanda, followed by other neighbouring countries (Uganda and Tanzania). The emergence of partial artemisinin resistance in the region is crucial because of its potential to spread to different parts of Africa, considering the Horn of Africa, which borders EA, has also recorded partial artemisinin resistance [7].

The official use of ACTs as a first-line treatment in the East African region began between 2004 and 2006 due to confirmed widespread resistance to CQ and SP [8–11] whereby Artemether-lumefantrine (AL) and artesunate-amodiaquine were adopted as the first-line treatments for uncomplicated *P. falciparum* malaria. Other ACTs, such as dihydroartemisinin-piperaquine, are recommended as alternative or second-line treatments [12].

ACTs were adopted in EA due to their high safety and efficacy against multidrug-resistant *P. falciparum* strains according to the World Health Organisation (WHO) recommendation [13], their capability for rapid reduction of parasite biomass, and the elimination of remnant parasites, thus lowering the risk of recrudescence [8–11,14]. Unfortunately, partial artemisinin resistance has been identified after a decade of ACT use in the region, particularly Rwanda, Tanzania and Uganda [3,6,15–18].

Ariey et al. [19] demonstrated the occurrence and role of key mutations in the *Pf*-*Kelch13* propeller gene (Pf3D7_1343700) in mediating delayed parasite clearance following ACT treatment in SEA [20]. Since then, more than 200 non-synonymous *Pf*-*Kelch13* mutations in the propeller domain have been documented worldwide; however, only a few are associated with artemisinin resistance [21]. The WHO classifies these mutations as validated (confirmed to delay parasite clearance *in vivo* and *in vitro*) and candidate (observed to cause delayed clearance in *vivo* but not in *vitro*) [22].

As stated earlier, the *Pf-Kelch13* mutations identified in East Africa have been confirmed to be indigenous (do not relate to those reported in SEA) [3,6], increase at a high rate, and have a high spreading capacity [15–17], making antimalarial drug resistance in the region an urgent threat to the global fight against malaria. Worse still, the Horn of Africa, which is a neighbouring region to East Africa, has also confirmed partial artemisinin resistance [23]. Hence, there is a potential for the spread of artemisinin resistance to countries neighbouring or interacting with the East African region. If not contained, partial artemisinin resistance may be followed by partner drug resistance, resulting in clinical treatment failure and total ACT resistance, which could jeopardise the global efforts towards malaria elimination.

As countries in the East African region strive to reduce the burden and eliminate malaria, understanding the patterns and varieties of artemisinin resistance molecular markers, particularly *Pf*-*Kelch13,* is imperative as part of the WHO strategy to combat antimalarial drug resistance in Africa [1]. In this review, we examine studies that document *Pf-Kelch13* mutations across East African countries to determine the magnitude of non-synonymous mutations mediating artemisinin resistance, their types, and the impact on treatment outcomes (day-3 parasitemia).

## Methods

The protocol for developing this systematic review and meta-analysis was registered on PROSPERO (Reference number: CRD42024602752). To select studies for inclusion in this review, we employed the Preferred Reporting Items for Systematic Review and Meta-Analysis Protocols (PRISMA-P) 2020 checklist [24]: PRISMA statement (prisma-statement.org).

### Information source and search strategy

A literature search for published studies assessing artemisinin resistance, particularly the *Pf-Kelch13* mutation among malaria patients in East Africa, was conducted using the Cochrane Central Register of Controlled Trials (CENTRAL), EMBASE, Google Scholar, Medline, and LILACS online databases. References were cited and managed using EndNote software version X8.

The measure of effect was the proportion of samples from malaria patients with non-synonymous *Pf-Kelch13* mutations mediating artemisinin resistance, the type of non-synonymous mutations, and the proportion of patients with parasitemia after day-3 of treatment.

The primary searching keywords used were “magnitude”; “prevalence”; “proportion”; ‘burden”; “artemisinin resistance”; “*Kelch13*”; “*K13*”; “malaria patients”; Boolean operator terms were searched separately and together accordingly as “OR” or “AND” or AND NOT or AND, NOT. The search included all articles published until December 2025.

### Data extraction

The results of the literature search were screened by two independent reviewers. Studies were selected by the reviewers according to the inclusion criteria. A third reviewer was consulted when there were differences in opinion between the two reviewers on the inclusion and methodological quality of the studies. Abstracted information /data was entered into the extraction sheet. The basic information extracted included the general information (author’s names, country in which the study was carried out, samples collection year and publication year), study characteristics (study design, sample size, study participants), the total number of samples genotyped, the proportion of samples with non-synonymous *Pf*- *Kelch13* mutation mediating artemisinin resistance, type of non-synonymous mutation and the proportion of patients with parasitaemia after day-3 of treatment.

### Study inclusion criteria

All published studies on *Pf*-*Kelch13* resistance markers were screened. We included studies that collected samples between 2014 and 2024. The year 2014 was used as a reference since the *Pf-Kelch13* mutation was first reported in East Africa, specifically in Rwanda, that year [3,25]. These studies consisted of original research articles published in peer-reviewed journals and were conducted exclusively in East Africa.

### Study exclusion criteria

To be specific to *Plasmodium* non-synonymous *Pf-Kelch13* mutations established to mediate artemisinin resistance, our review did not include studies reporting non-synonymous mutations not mediating artemisinin resistance, only synonymous *Pf*-*Kelch13* mutations or those that describe other markers of antimalarial drug resistance (*Pfcrt, Pfmdr1, Pf* coronin, *Pf-*ATPase6, plasmepsin genes, cysteine desulfurase, *Pf-Kelch12*, and other *P. falciparum* resistance genes), as meta-analyses for these genes have been published elsewhere or fall outside the scope of this review. Studies detailing mutations in other Plasmodium species were also excluded. Additionally, studies reporting *Pf*-*Kelch13* mutations before 2014 were excluded.

Since the aim of this review was to establish the proportions of non-synonymous *Pf*-*Kelch13* mutations mediating artemisinin resistance and their influence on treatment outcomes, particularly day-3 parasitaemia among malaria patients in East Africa, where partial artemisinin resistance has been identified, studies reporting treatment outcomes including day-3 parasitaemia among malaria patients without information on *Pf*-*kelch13* mutation were excluded.

Literature reviews, non-primary research studies, and conference abstracts were considered to lack sufficient information or reliable sources and were thus excluded from the present review.

### Methodological and data quality assessment

A methodological quality assessment was conducted employing the National Institutes of Health (NIH) study quality assessment tools for controlled intervention studies, observational cohorts, and cross-sectional studies [26]. The score range for the NIH tool was 0–14, with each criterion worth 1 point, for a total of 14 points. The scores were converted into percentages, which were categorised as 0–60% (Poor), 61–80% (Fair), and 81–100% (Good) [26]. All articles were of good quality. Two independent reviewers reached consensus on the extracted data and the methodological quality assessment. The scores of the included studies are presented in Table 1.

**Table 1:** Study characteristics.

No	Country	Authors	Year Publication	Study type	Sample size	Sample collection year	Sample type	Method for K13 detection	Population	Transmission	Score	Reference
1	Rwanda	Tacoli et al.	2016	Cross-sectional	222	2010-2015	Blood	Sequencing	Malaria-infected children	Low	93	[29]
2	Uganda	Asua et al.	2019	Cross-sectional	412	2017	Blood	Sequencing	Malaria-infected children	Not stated	100	[30]
3	Uganda	Ikeda et al.	2018	Cross-sectional	194	2014-2016	DBS	Sequencing	Malaria-infected patients	High	93	[31]
4	Uganda	Balikagala et al.	2021	Longitudinal	240	2015 −2019	DBS	Sequencing	Malaria-infected patients	High	100	[15]
5	Rwanda	Uwimana et al.	2021	Open-label, single-arm trial	228	2018	DBS	Sanger sequencing	Children aged 6–59 months with UCMA	Low	100	[17]
6	Rwanda	Uwimana et al.	2020	Clinical drug efficacy studies	534	2013 −2015	Blood	Sanger Sequencing	Patients with UCMA	Not stated	86	[3]
7	Rwanda	Straimer J, et al.	2021	Multicenter, randomised, open-label	73	2018- 2019	Blood	Sequencing	Adults with UCMA	Not stated	100	[32]
8	Tanzania	Ishengoma et al.	2019	Single-arm prospective in vivo	344	2016	DBS	Sanger sequencing	Children 6 months to 10 years with UCMA	Low	100	[33]
9	Uganda	Kamilo et al.	2024	Prospective longitudinal study	100	2022	Blood	Sanger Sequencing	Malaria patients on ALU	Over 50%	100	[34]
10	Kenya	Maniga et al.	2023	Cross-sectional health point prospective	231	2021	Blood & DBS	Sanger sequencing	Malaria-infected patients	Not stated	93	[35]
11	Rwanda	Bergmann et al.	2021	Therapeutic efficacy study	76	2019	Blood	Sequencing	Patients with UCMA	Not stated	93	[36]
12	DR Congo	Yobi et al.	2021	Therapeutic efficacy study	364	2018–2019	DBS	Sanger-sequencing	Treated patients returning with fever	Not stated	86	[37]
13	DR Congo	Kahunu et al.	2024	Therapeutic efficacy study	1,115	2020–2021	DBS	Sequencing	Children aged 6–59 months with UCMA	Not stated	93	[38]
14	Uganda	Awor et al.	2024	Cross-sectional	697	2018-2020	DBS &Blood	Whole genome sequencing	Children below 5 years with severe malaria	Not stated	93	[6]
15	Rwanda	Kirby et al.	2023	Cross-sectional	1873	2014-2015	DBS	Sequencing	women and children	Not stated	86	[25]
16	Kenya	Jeang et al.	2024	Longitudinal cohort	775	2018-2022	DBS	Sanger sequencing	children 5–18 years	Low, holoendemic, mesoendemic, hypoendemic	93	[39]
17	Kenya	Akala et al.	2024	Therapeutic efficacy study	679	2018-2024	Blood	Illumina sequencing	6 months and above with UCMA	Not stated	93	[40]
18	DR Congo	Moriarty et al.	2021	In vivo therapeutic efficacy studies	486	2017	DBS	Sanger sequencing	Children aged 6–59 months with UCMA	Varying intensity	93	[41]
19	Tanzania	Mhamilawa et al.	2020	Randomised controlled, parallel group, superiority clinical trial	280	2017	DBS	Sequencing	Adults and Children	Moderate	100	[42]
20	Tanzania	Laury et al.	2025	Open-label prospective	352	2022	DBS	Sanger-sequencing	6 months to 10 years	Not stated	93	[43]
21	Tanzania	Msellem et al.	2020	Single-armed therapeutic efficacy trial	142	2017	DBS	Sanger sequencing	All ages	Not stated	100	[44]
22	Tanzania	Bakari et al.	2024	Therapeutic efficacy study	1769	2016-2021	DBS	Capillary sequencing	6 months to 10 years	Varying intensity	86	[45]
23	Tanzania	Ishengoma et al.	2024	Single-arm therapeutic efficacy study	6855	2021	DBS	Whole genome sequencing	6 months to 120 months	Not stated	100	[18]
24	Uganda	Asua et al.	2021	Cross-sectional	796	2018-2019	DBS	Next-generation Sequencing	>6 months- 10 years of age (for 2018), all ages (for 2019)	Varying intensity	93	[46]

### Data collection and analysis

The proportions of non-synonymous *Pf*-*Kelch13* mutations mediating artemisinin resistance, along with the types and proportions of treated patients exhibiting day-3 parasitaemia among malaria patients, were recorded in an Excel sheet. The proportion of *Pf-Kelch13* mutation is defined as the proportion (%) of patients with the mutation confirmed by molecular techniques. Meta-analyses were conducted using STATA 17 (Statistical Corporation, College Station, TX, US). A random effects model was employed to consolidate information from comparable studies. The potential for publication bias was evaluated by examining asymmetry on funnel plots. Heterogeneity across studies was assessed using Cochran’s Q and I^2^. Heterogeneity was deemed substantial when the p-value of Q was < 0.10, or I^2^ exceeded 50% [27]. A sensitivity analysis was performed to examine the influence of each study on the overall proportion estimation through leave-one-out meta-analysis [28]. Ineligible articles and duplicates were excluded from the analysis. The pooled proportions/estimates of samples from malaria patients with non-synonymous *Pf*-*Kelch13* mutations and day-3 parasitaemia among those treated with ACTs were illustrated using forest plots. Subgroup analyses were conducted to present the aggregated proportions for the types of mutations, year of sample collection and within each country.

## Results

### Study attributes

**Fig 1:**
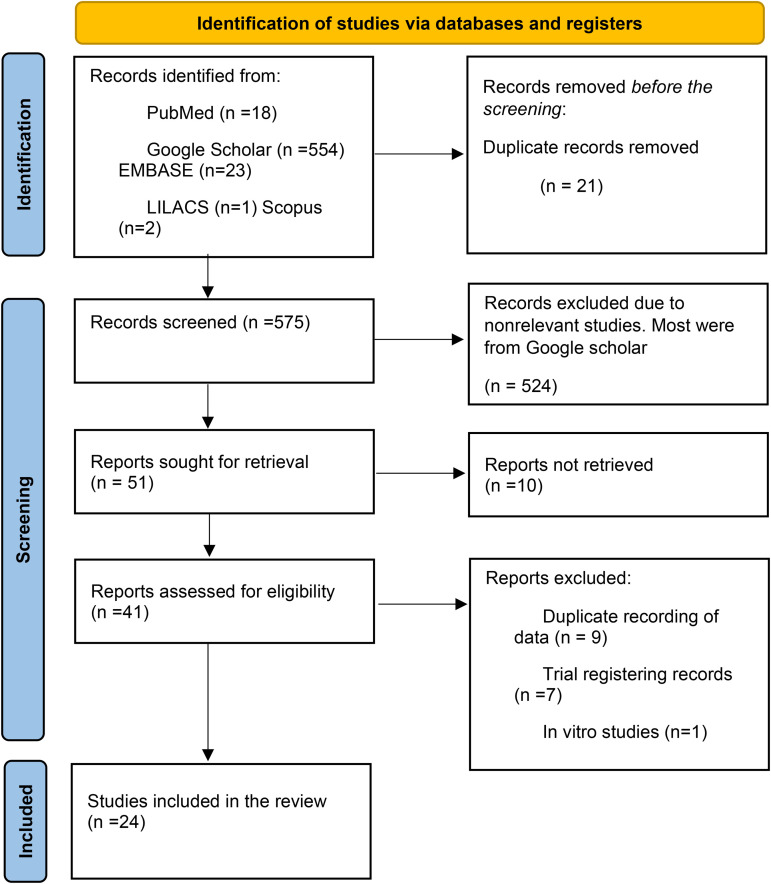
PRISMA flow chart illustrating the article selection steps [20].

The electronic database search identified 575 records reporting SNPs in the *Pf-Kelch13* gene, as shown in the PRISMA flow chart (Fig 1). These articles were included for full-text review. A total of 24 studies were eligible for data extraction and were therefore included in the meta-analysis for the estimation of the pooled proportions of *Pf*-*Kelch13* non-synonymous mutations. These studies were conducted in five East African countries (Uganda, Rwanda, the Democratic Republic of Congo, Tanzania, and Kenya). Two countries (Burundi and South Sudan had no eligible studies for data extraction. A total of 15,951 *P. falciparum* malaria patients provided isolates for this review. Studies included were published between 2016 and 2025, while samples for these studies were collected from 2010 to 2024. Most of the studies (60%) employed samples from dried blood spots (DBS). Details on the study characteristics are provided in Table 1.

### Heterogeneity, publication bias, and sensitivity analysis

**Fig 2:**
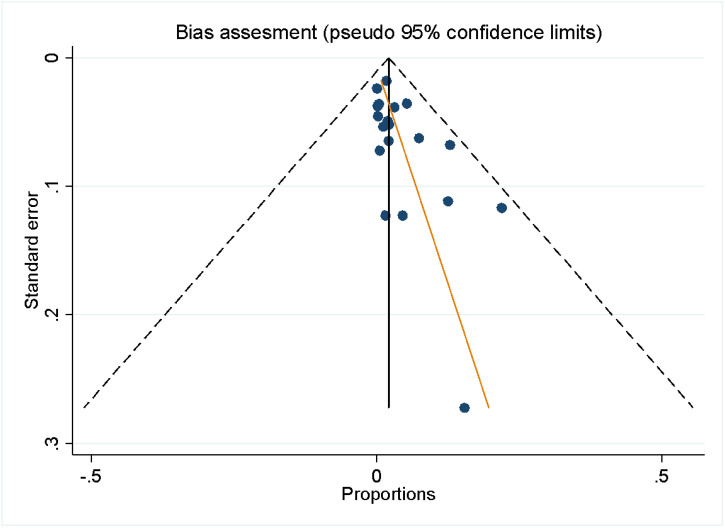
Funnel plot for the determination of pooled proportions of *Pf-Kelch13* non-synonymous mutations among malaria patients. The dashed lines represent the 95% confidence interval (CI). The X-axis represents the proportions, and the dots show the distribution of individual studies.

The heterogeneity among the studies included in this meta-analysis was substantial (I^2^ > 95% and p < 0.01). The funnel plot was symmetrical, indicating that the conducted meta-analysis was not influenced by publication bias, as illustrated in Fig 2. A sensitivity analysis demonstrated robustness of the aggregated estimates (S3 Fig).

## Discussion

This review evaluates various non-synonymous mutations of the *Pf*-*Kelch13* gene in the East African region, where partial artemisinin resistance has been reported specifically in Uganda, Tanzania and Rwanda [3,15,18].

**Fig 3:**
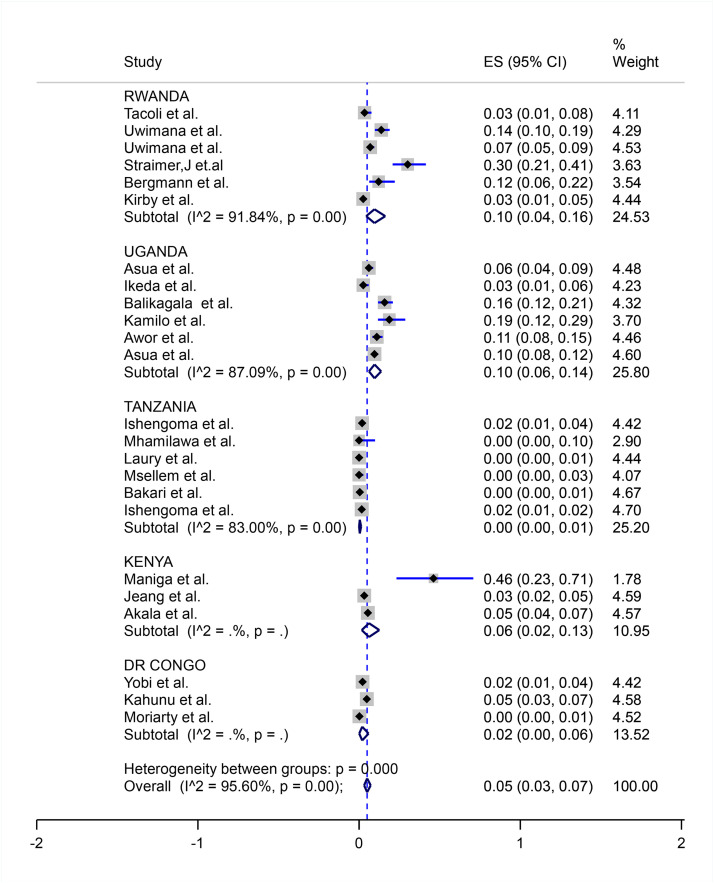
Proportion of non-synonymous *Pf-Kelch13* mutations mediating artemisinin resistance in East Africa.

A substantial prevalence of *Pf-Kelch13* non-synonymous mutations associated with artemisinin resistance reported in this review varies between countries. The highest prevalences (≥10%) of these mutations were observed in Uganda and Rwanda (Fig 3). The considerable prevalence of non-synonymous mutations confirmed to mediate artemisinin resistance poses a challenge to the efficacy of ACT and treatment outcomes in the region.

**Fig 4:**
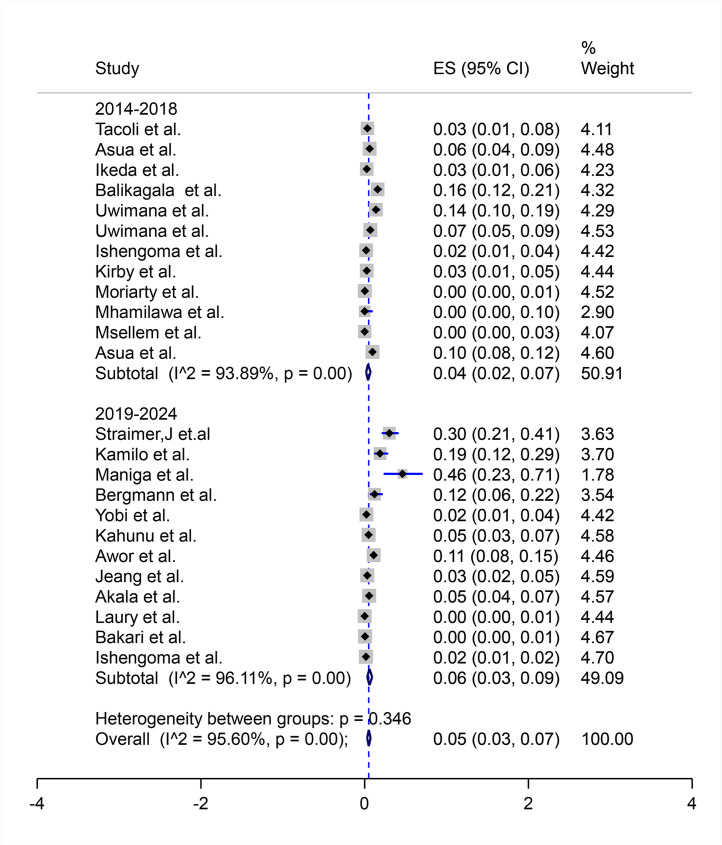
Proportion of non-synonymous *Pf-Kelch13* mutation mediating artemisinin resistance in East Africa (ten-year span).

The *Pf-Kelch13* non-synonymous mutations have been slightly increasing during the ten years of the study (Fig 4). This is supported by recent studies, which have suggested that the indigenous mutations in East Africa are spreading at a high capacity [15–17]. The prevalence of these mutations may differ (high) at present, considering we only report studies which involve samples collected up to 2024. This calls for enhanced efforts to mitigate the emergence and spread of resistance in the region.

In general, the pooled prevalence reported in the region is lower, contrary to findings from SEA, where the aggregated prevalence of 45.5% has been observed, but presents with less diversity compared to East Africa [20]. This discrepancy is not surprising, considering that the *Pf-Kelch13* mutations reported in East Africa are indigenous and have been identified recently compared to SEA. The variations in the introduction time of artemisinin between SEA and East Africa (1970s vs 2000s) [8–11,47] could also explain the difference in the frequencies of non-synonymous mutations between the two regions, whereby the higher drug pressure is observed with SEA than in East Africa; thus, parasite selection is higher in SEA [48].

**Fig 5:**
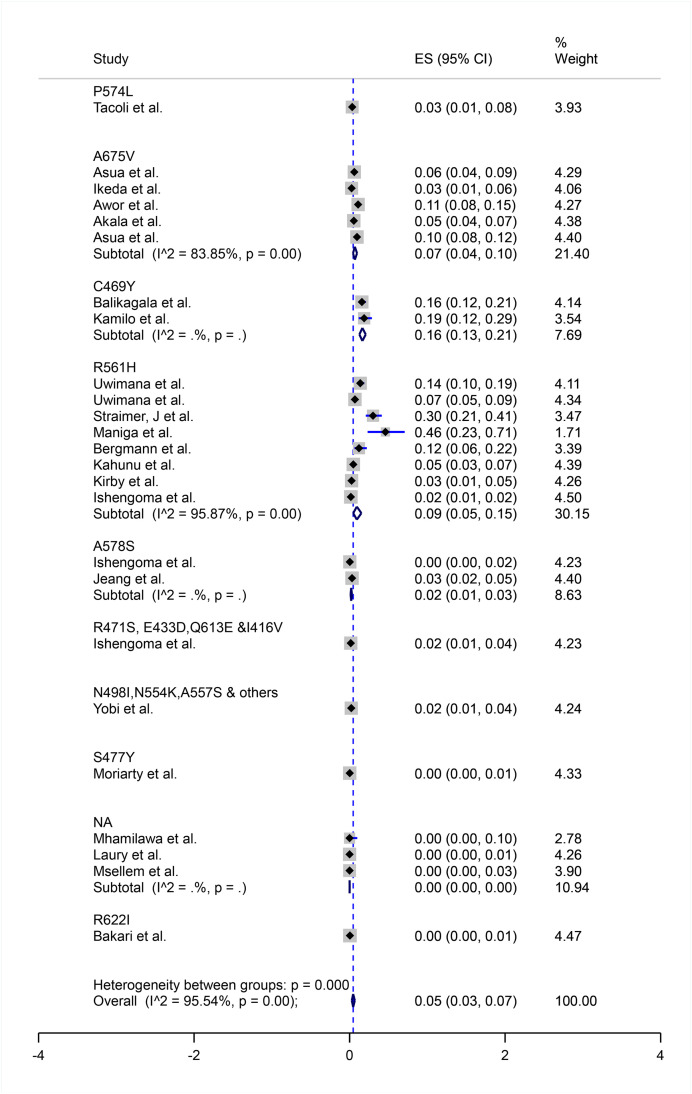
Proportion for types of non-synonymous *Pf-Kelch13* mutation mediating artemisinin resistance in East Africa. Note: NA: Not applied (for those with zero non-synonymous mutations); Others: refers to non-synonymous mutations which had not yet been reported elsewhere.

The most prevalent *Pf*-*Kelch13* non-synonymous mutation mediating artemisinin resistance in East Africa was R561H (Fig 5). R561H was reported in Uganda, Kenya, Tanzania, Rwanda, and DR Congo (five countries). The mutation was high in the bordering regions between them, suggesting there is a spread of the *Pf-Kelch13* mutant gene/SNPs as a result of population interaction/migration due to socio-economic reasons. This is echoed by the evidence from Uganda, which has shown that the selection of *Pf-Kelch 13* is similar to that in SEA, hence a quick increase and high spreading potential to other parts [49].

The A675V was the next most prevalent *Pf-Kelch13* non-synonymous mutation in the East African region, existing at higher frequencies in Uganda and Kenya. Other *Pf-Kelch13* non-synonymous mediating artemisinin resistance mutations have been reported in East Africa, albeit at low frequencies. These include C469Y, which was prevalent in Uganda and DR Congo [50], while R622I was identified in Tanzania and Kenya*.* The P441L, which has only been reported in Africa, was identified in DR Congo [38]. A578S, which is most prevalent in Africa [51], though some studies report its lack of association with artemisinin resistance [51,52], was not common in East Africa. However, this mutation is reported to be associated with low parasitaemia in Uganda [53]. Hence, this study included this marker.

The emergence of the non-synonymous mutations and partial artemisininresistance in the East African region may be attributed to the improper or irrational use of antimalarials through self-medication and inappropriate prescriptions, lack of adherence to guidelines, and the circulation of counterfeit or poor-quality antimalarials in the community [38,54–56].

The use of monotherapy in the early 2000s across all countries [57], before the shift to ACTs, along with the continued use of these treatments for uncomplicated malaria (not outlined in the treatment policy or guidelines) in some countries, may have contributed to the selection of artemisinin resistance in the region [16,21]. Moreover, the use of parenteral artesunate and artemether (reserved for severe malaria) to treat uncomplicated cases may also facilitate the emergence of resistant parasites in the communities [58].

It is worth noting that the Denovo *Pf*-*Kelch13* mutations have now spread to various countries in East Africa and could extend to other sub-Saharan nations. As stated earlier, the region is a historical hotspot for the spread of antimalarial resistance across the continent [4,5]; therefore, the emergence and propagation of partial artemisinin resistance [3,59] necessitate coordinated regional and global efforts to prevent the further spread of artemisinin resistance across the continent.

Recent findings suggest that the partial artemisinin resistance in most areas in East Africa has emerged in high transmission zones (where population immunity is high) [15], unlike the known trend for antimalarial resistance, where emergence is from low transmission zones, as seen in southern Africa [60,61], where low immunity in the population favours resistant parasites to survive. The reasons for this discrepancy are yet to be established.

**Fig 6:**
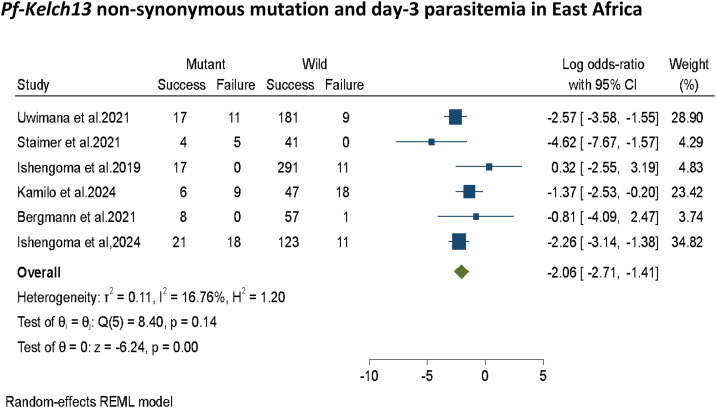
The odds for treatment failure (day-3 parasitaemia) in patients with non-synonymous mutations and wild-type *P. falciparum* in East Africa.

In this study, we evaluated research reporting parasite clearance among malaria-infected individuals with *Pf*-*Kelch13* non-synonymous mutations upon enrolment for treatment with ACTs. Patients harbouring *Pf-Kelch13* non-synonymous mutations were more likely to have day-3 parasitaemia than those with wild parasites after treatment with ACTs (Fig 6). This further confirms the presence of partial artemisinin resistance in the region.

Despite the efficacy of ACTs in the treatment of uncomplicated malaria in East African countries over recent years remaining at more than 96% [62–65]. It is worth noting that some areas in Rwanda, DR Congo, Tanzania and Uganda have reported artemether-lumefantrine failure above the WHO threshold for treatment change (10%) [12].

The emergence of artemisinin partial resistance calls for coordinated efforts among East African countries to systematically implement surveillance of parasite population genetics to ascertain their genetic origin and spreading potential, enabling timely interventions to mitigate the spread, improve case management, optimise resource allocation, and promote malaria elimination. This implies a need to scale up therapeutic efficacy studies assessing molecular markers for artemisinin resistance across entire regions in countries, rather than relying on data from a few selected sentinel sites in each country. Partner drug resistance markers surveillance is also warranted to avoid the emergence of total ACT failure in the region, taking into account the trend observed in SEA [66,67].

The present review has limitations; the data for analysis represent five countries, while two countries (Burundi and South Sudan) are not represented due to a lack of adequate evidence. Molecular surveillance on *Pf-Kelch13* is scanty in these countries. Moreover, some studies overlook key variables, including sample amplification rate, *Pf-Kelch13* prevalence, day-3 parasitaemia, and other treatment outcome parameters among patients harbouring *Pf-Kelch13* mutations (only a few studies have assessed the clinical impact of the recorded *Kelch-13* mutations). Studies looking at ex vivo data were also not included. The high heterogeneity recorded in the meta-analysis could affect the pooled estimates. The high heterogeneity could be attributed to differences in study design (S1 Fig), variation in sample size (S2 Fig), and study participants. Despite these constraints, this review is the first to provide evidence on the magnitude of non-synonymous *Pf*-*Kelch13* mutations mediating artemisinin resistance in the East African region.

**Conclusion:** Evidence provided by this review on the magnitude and types of *Pf-Kelch13* non-synonymous mutations confirmed to be associated with delayed parasite clearance calls for coordinated efforts to contain the partial artemisinin resistance in the region and the continent at large.

## Supporting information

S1 Fig Pooled proportions for *Pf-Kelch13* non-synonymous mutations mediating artemisinin resistance per type of study. (DOCX)

S2 Fig Pooled proportions for *Pf-Kelch13* non-synonymous mutations mediating artemisinin resistance per sample size. (DOCX)

S3 Fig Sensitivity analysis for the prevalence of *Pf-Kelch13* non-synonymous mutations mediating artemisinin resistance. (DOCX)

S1 File PRISMA 2020 Checklist. (DOCX)

## Abbreviations

ACT: artemisinin-based combination therapy
AL: artemether-lumefantrine
CQ: chloroquine
NIH: National Institutes of Health
*pfcrt*: *Plasmodium falciparum chloroquine transporter*
*PfK13*: *Plasmodium falciparum Kelch 13*
*pfpm2/3*: *Plasmodium falciparum plasmepsin 2/3*
SEA: South East Asia
sSA: sub-Saharan Africa
SP: Sulfadoxine-Pyrimethamine
WHO: World Health Organisation
