# Supplementary material for: Plasmodium falciparum non-synonymous Kelch13 mutations mediating artemisinin resistance in East Africa: A systematic review and meta-analysis: 2014–2024
Source: PLoS One. 2026 Jul 28;21(7):e0354429. doi: 10.1371/journal.pone.0354429 (PMC13411923; doi:10.1371/journal.pone.0354429)
Supplement: S2 Fig — (DOCX) [file pone.0354429.s002.docx]

**S2 File.**  Pooled proportions for *Pf-Kelch13* non-synonymous mutations mediating artemisinin resistance per sample size
