# Supplementary material for: Plasmodium falciparum non-synonymous Kelch13 mutations mediating artemisinin resistance in East Africa: A systematic review and meta-analysis: 2014–2024
Source: PLoS One. 2026 Jul 28;21(7):e0354429. doi: 10.1371/journal.pone.0354429 (PMC13411923; doi:10.1371/journal.pone.0354429)
Supplement: S3 Fig — (DOCX) [file pone.0354429.s003.docx]

**S3 File.**  Sensitivity analysis for the prevalence of *Pf-Kelch13* non-synonymous mutations mediating artemisinin resistance.

**NB:** 1= Tacoli et al.; 2= Asua et al.;3= Ikeda et al.;4=Balikagala et al.;5=Uwimana et al.;6=Uwimana et al.;7=Straimer,J et al.;8=Ishengoma et al.;9=Kamilo et al.;10-=Maniga et al.;11=Bergmann et al.;12=Yobi et al.;13=Kahunu et al.;14=Awor et al.;15=Kirby et al.;16=Jeang et al.;17=Akala et al.;18=Moriarty et al.;19=Mhamilawa et al.;20=Laury et al.;21=Msellem et al.;22=Bakari et al.;23=Ishengoma et al.24=Asua et al.
